# Dr. Samuel Griffith

**Published:** 1859-09

**Authors:** 


### NECROLOGICAL NOTICES.

Dr. Samuel Griffith.—This young and estimable physician died at Devon, on the 23d of June, in the thirty-fourth year of his age. Enjoying one of the largest practices in the diseases of females, he overtasked his constitution, which brought on disease of the lungs, eighteen months prior to his death. Dr. Griffith was a zealous student, and at the time of his decease he occupied many important positions, among which was that of Physician-Accoucheur, and Lecturer on Clinical Midwifery, to St. Thomas's Hospital.

